# Disrupting the circadian clock: Gene-specific effects on aging, cancer, and other phenotypes

**DOI:** 10.18632/aging.100323

**Published:** 2011-05-01

**Authors:** Elizabeth A. Yu, David R. Weaver

**Affiliations:** Department of Neurobiology, MD/PhD Program and Program in Neuroscience, University of Massachusetts Medical School, Worcester, MA 01605, USA

**Keywords:** circadian rhythms, clock, Bmal1, period, cryptochrome, cancer, aging

## Abstract

The circadian clock imparts 24-hour rhythmicity on gene expression and cellular physiology in virtually all cells. Disruption of the genes necessary for the circadian clock to function has diverse effects, including aging-related phenotypes. Some circadian clock genes have been described as tumor suppressors, while other genes have less clear functions in aging and cancer. In this Review, we highlight a recent study [Dubrovsky et al., *Aging* 2: 936-944, 2010] and discuss the much larger field examining the relationship between circadian clock genes, circadian rhythmicity, aging-related phenotypes, and cancer.

## INTRODUCTION

In a recent issue of Aging, Dubrovsky et al. [1] describe the effects of disruption of the Clock gene on lifespan and health in mice. They report that CLOCK-deficient mice have reduced average and maximum lifespan, and have increased incidence of dermatitis and cataracts as the animals age [1]. Notably, however, targeted disruption of the Clock gene does not lead to the same constellation of phenotypes as seen in mice with disruption of other genes critical for circadian clock function (Tables 1 and 2). In this Review, we summarize and discuss potential reasons for the gene-specific effects of circadian clock gene disruption. Why do these mouse models differ? And, what do these differences tell us about the role of the circadian clock in the regulation of physiology, healthy aging, and responses to genotoxic stress? Before addressing these questions, we will describe the circadian clock mechanism and the circadian clock gene hypothesis of cancer/aging.

**Table 1.** Rhythm and aging phenotypes of mice with mutations in genes contributing to the transcriptional activator complex

	*Clock^Δ^^19/^^Δ^^19^*	*Clock^−/−^*	*Npas2^m/m^*	*Clock^−/−^;Npas2^m/m^*	*Bmal1^−/−^*
**Circadian Behavior:**					
Rhythmic in DD?	Varies [123,141-144]	Yes [4, 117]	Yes [4, 145]	No [4]	No [106]
Period length	Long [~28 hr] to AR	Slightly shorter	Slightly shorter	N/A - Arrhythmic	N/A - Arrhythmic
***Ex vivo* Rhythms:**					
SCN slice	Arrhythmic [124]	Rhythmic [4, 118]	Rhythmic [118]	Arrhythmic [118]	Arrhythmic [115, 124; but see 147]
Peripheral tissues	Arrhythmic [115, 124]	Arrhythmic [118]	Rhythmic [118]	Arrhythmic [118]	Arrhythmic [115, 147]
**Aging Phenotypes**					
Lifespan	Reduced (Female) Normal (Male) [49]	Reduced (Both) [1]	Seems OK (Both) [our unpublished]	Reduced (Both) [116]	Reduced (Both) [50, 107, 111]
Body weight	Increased [148]	Normal [1]	N.D.	Reduced [our unpublished]	Reduced [50]
Organ weights	Normal [148]	Normal [1]	N.D.	N.D.	Most Reduced [50]
Cataract incidence	N.D.	Increased [1]	N.D.	N.D.	Increased [50]
Dermatitis	N.D.	Increased [1]	N.D.	N.D.	Normal [48]
Ectopic calcification	No [our unpublished]	No [116]	No [116]	Yes [116]	Yes [108, 112]
Reproduction	Modest decrease [121, 122, 150-153]	Modest decrease [our unpublished]	Seems OK [4, 145, our unpublished]	Sterile [our unpublished]	Sterile [109, 110]
Tumor incidence	Normal [49]	N.D.	N.D.	N.D.	N.D.
**Response to Irradiation**					
Lifespan	Reduced (Female) Normal (Male) [49]	N.D.	N.D.	N.D.	N.D.
Body weight	Reduced (Both) [49]	N.D.	N.D.	N.D.	N.D.
Organ weights	Some Reduced [F]; Normal (Male) [49]	N.D.	N.D.	N.D.	N.D.
Cataract incidence	Increased (Both) [49]	N.D.	N.D.	N.D.	N.D.
Dermatitis	N.D.	N.D.	N.D.	N.D.	N.D.
Tumor incidence	Normal [49]	N.D.	N.D.	N.D.	N.D.

**Table 2.** Rhythm and aging phenotypes of mice with mutations in genes contributing to the circadian repressor complex

	*Cry1^−/−^*	*Cry2^−/−^*	*Cry1^−/−^; Cry2^−/−^*	*Per1^−/−^*	*Per2^m/m^*	*Per1^−/−^; Per2^m/m^*
**Circadian behavior:**						
Rhythmic in DD?	Yes [7,	Yes [7, 8]	No [7, 8]	Varies [6, 9, 154]	Varies [6, 9, 156]	No [6, 9]
Period length	Shorter	Longer	N/A - Arrhythmic	Short or Normal	Short to Normal	N/A - Arrhythmic
***Ex vivo* Rhythms:**						
SCN slice	Rhythmic [146]	Rhythmic [146]	Arrhythmic [146]	Rhythmic [146] Arrhythmic [155]	Rhythmic [158]	N.D.
Peripheral tissues	Arrhythmic [146]	Rhythmic [146]	Arrhythmic [146]	Arrhythmic [146]	Varies [158]	N.D.
**Aging parameters:**						
Lifespan	N.D.	N.D.	N.D.	N.D.	Decreased	N.D.
Body weight	N.D.	Normal [162]	Decreased [159] Normal [163]	Decreased [160, 161]	Increased [160, 161]	Normal [163]
Organ weights	N.D.	N.D.	N.D.	N.D.	N.D.	N.D.
Cataract incidence	N.D.	N.D.	N.D.	N.D.	N.D.	N.D.
Dermatitis	Increased [48]	N.D.	Increased [48]	N.D.	Normal [48]	Normal [48]
Ectopic calcification	No	No	No	No	No	No
Bone mass	N.D.	Increased [162]	Increased [163]	Normal [163]	Normal [163]	Increased [162, 163]
Reproduction	Seems OK (Both) [our unpublished]	Seems OK (Both) [our unpublished]	Seems OK (Both) [our unpublished]	Seems OK (Male) [our unpublished] Reduced (Female) [161]	Seems OK (Male) [our unpublished] Reduced (Female) [161]	Seems OK (Male) [our unpublished] Reduced (Female) [our unpublished]
Tumor incidence	Increased [48]	N.D.	Normal [23] Increased [48]	N.D.	Increased [46, 48]	Increased [48]
**Response to Irradiation**						
Lifespan	N.D.	N.D.	Normal [23] Reduced [48]	N.D.	Reduced [46, 48]	Reduced [48]
Body weight	N.D.	N.D.	N.D.	N.D.	N.D.	N.D.
Organ weights	N.D.	N.D.	N.D.	N.D.	N.D.	N.D.
Cataract incidence	N.D.	N.D.	N.D.	N.D.	N.D.	N.D.
Dermatitis	Small Increase [48]	N.D.	Increased [48]	N.D.	Normal [48]	Increased [48]
Tumor incidence	Increased [48]	N.D.	Normal [23] Increased [48]	N.D.	Increased [46,48]	Increased [48]
**Cancer-prone model**			***p53^−/−^***		***APCmin/+***	
Cancer incidence	N.D.	N.D.	Decreased [51]	N.D.	Increased [47]	N.D.
Lifespan	N.D.	N.D.	Increased [51]	N.D.	Decreased [47]	N.D.

### The circadian clock is based on a transcriptional-translational feedback loop

**Figure 1.**
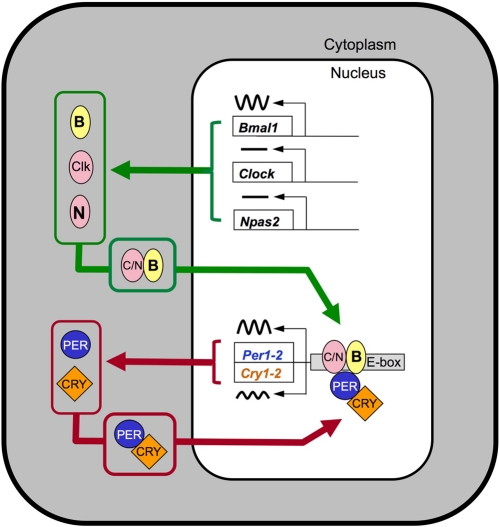
Model illustrating the core transcriptional-translational feedback loop underlying circadian rhythmicity. The components of the transcriptional activator complex, CLOCK, BMAL1, and (within SCN) NPAS2 form heterodimers that activate transcription of E-box containing genes, including the negative regulatory PER and CRY proteins. The PER-CRY protein complexes accumulate in the nucleus and block the activity of the activator complex. The alternation between the activation phase and the repression phase has a cycle length of near 24 hours. For simplicity, key events including posttranslational modifications and the generation of cascades of transcription factors leading to rhythmicity of tissue-specific “output” genes are not shown. For a more detailed model, see [2].

Many cell types contain cell-autonomous circadian clocks that measure 24 hours. These clocks impart rhythmicity on function in virtually every organ. The biological timekeeping mechanism is based on a negative feedback loop involving the rhythmic production, followed by protracted degradation, of protein complexes that shut off their own production. The alternation between transcriptional activation and transcriptional inhibition occurs with a cycle length of approximately 24 hours [for review see 2,3]. In mammals, at the core of this mechanism are genes called Clock and Bmal1 (also called Mop3 or Arntl) (Figure 1). The protein products of these genes, CLOCK and BMAL1, are basic helix-loop-helix-PAS (bHLH-PAS) domain-containing transcription factors. They are dimerization partners of central importance to the function of the circadian clock, although NPAS2, another bHLH-PAS protein, can substitute for CLOCK as partner for BMAL1 in neurons of the suprachiasmatic nucleus (SCN) that control locomotor activity rhythms [4,5]. Proteins critical for closing the negative feedback loop include the products of the Per1 and Per2 genes, and the Cry1 and Cry2 genes; within these gene families there is redundancy of function, such that disruption of both Per1 and Per2, or both Cry1 and Cry2, is necessary to completely disrupt circadian rhythmicity [6-9]. Because these mutant lines provide multiple ways to disrupt circadian clock function, one might expect that a coherent picture of the role of the circadian clock in aging and the response to genotoxic stress would result. However, these mutant lines differ in their aging-related phenotypes, as well as having subtle differences in their circadian clock function.

### The circadian clock gene hypotheses of cancer and aging, corollaries and caveats

A great deal of experimental effort has addressed the role of the circadian clock genes in aging and especially in cancer-related phenotypes. The hypothesis being tested is usually that a circadian clock gene functions as a tumor suppressor gene, playing a role in retarding cancer development (or aging). More specifically, the hypothesis is that “circadian genes are involved in (cancer-related) biological pathways such as cell proliferation and apoptosis by controlling the expression of tumor suppressor genes, cell cycle genes, and genes encoding caspases and transcription factors” [10]. The basis for this hypothesis is that there are interactions between the cell cycle and circadian cycle, and dysregulation of the circadian clock is then thought to lead to dysregulation of cell cycle control [10].

Corollaries (with emphasis on cancer) are:

1. mutations of circadian clock genes will be associated with altered rates of cancer;
2. circadian “clock gene” and protein expression levels will be altered in tumors;
3. mutant mice with disrupted circadian clock genes will have altered (increased) cancer incidence under basal aging conditions, following gamma-irradiation, and in cancer-prone models.

Studies in support of this hypothesis are plentiful, as reviewed in greater detail below. Several studies indicate that circadian clock genes and their products contribute to regulation of cell cycle genes, apoptosis, and gene expression, and indicate molecular mechanisms by which these processes might be affected by clock gene products [11-21] [for recent reviews, see 22-33]. Many studies report altered (usually decreased) levels of clock gene expression in tumors [34-39; but see 40,41]. Conversely, in experimental models, overexpression of clock genes in tumors or in cancer cell lines reduces tumor growth [11, 42-45]. Mice homozygous for disruption of circadian clock genes that lead to loss of rhythmicity have many phenotypic abnormalities. Among these phenotypes are increases in basal cancer incidence or in the incidence of cancer following genotoxic stress or in genetically cancer-prone models [46-50; but see 51]. Similarly, knocking down the expression level of circadian clock genes can promote cancer growth [19, 52-55; but see 56, 57]. In humans, genome-wide association studies and more targeted studies have identified allelic variants, mutations, and epigenetic modifications that are associated with differences in risk of cancer [58-70]. Collectively, then, do these studies provide strong evidence that the circadian clock, and circadian rhythmicity, regulate aging and cancer? Remarkably, no. While the contribution of several circadian clock genes to cancer defense seem well-established, the contribution of the circadian clock is much less clear.

### Functional impact of mutant alleles

In epidemiological studies, the circadian clock gene hypothesis of cancer proposes that genetic variations in clock genes are likely to be associated with individual susceptibility to cancer [62]. A more specific formulation of the hypothesis is that “circadian disruption may be a novel risk factor and that genetic determinants for circadian rhythms may play a role in… tumorigenesis” [59], which emphasizes the interaction of genetic predisposition based in the circadian clock genes with environmental influences (especially shift work and light exposure at night, which can disrupt diurnal rhythms; see below). Several epidemiological studies have been conducted, examining allele frequencies for polymorphisms in and around clock genes in association with different types of cancer. Many positive associations have been reported, both in targeted analyses and in genome-wide association studies [58-70].

An important factor for interpreting epidemiological studies is the observation in mutant mice that null alleles of circadian clock genes are usually recessive; that is, animals heterozygous for a loss-of-function allele have little or no deficit in overt circadian rhythms. Furthermore, within several of the circadian gene families, there is functional redundancy between closely related genes [Reviewed in [2]]. Mutations that lead to loss of circadian clock function have global effects on systemic physiology and metabolism, endocrine function and behavior. The functional impact of single nucleotide polymorphisms (SNP's) within or near circadian clock genes on the maintenance of rhythmicity is much less clear. It is likely that most SNPs near circadian genes do not lead to demonstrable changes in circadian oscillator function, either centrally or peripherally. Circadian clock genes may be influencing disease susceptibility due to their pleiotropic activities on gene expression or involvement in other pathways, rather than through their involvement in circadian clock function. As with GWAS studies in most fields, progression from identification of loci to elucidation of cellular and biochemical mechanisms is quite difficult.

### Gene expression rhythmicity or gene expression level?

Microarray studies show that from 5-10% of transcripts are expressed with 24-hour rhythmicity. The specific genes that are rhythmic vary from tissue to tissue [17, 71-82], but key, rate-limiting steps are often rhythmic [71,72]. Disrupting critical circadian clock genes not only disrupts rhythmicity of target gene expression, but it can also have dramatic consequences on the level of expression of many target genes [73. 76-77]. Furthermore, the manner in which the circadian feedback loop is disrupted often has differing effects on the resulting level of gene expression. So, one might expect that disrupting genes on the activational side of the transcriptional feedback loop (Clock/Bmal1/Npas2) might have effects on target gene expression that are opposite to the effects seen when disrupting the genes whose products form the repressor complex (Pers and Crys; in this case the activator complex may act unopposed (Figure 1)). (To use a household analogy, in the former case the furnace is broken so no heat is produced; in the latter case the thermostat is broken so the signal to turn off heat production is not given, and the room overheats. In both cases the system is broken, but the effects on steady state room temperature are opposite). An interesting distinction to keep in mind when considering phenotypes resulting from circadian gene disruption is whether the phenotype could be due to loss of rhythmicity of gene expression, versus due to the potentially disruption-specific effects on gene expression level. In the former case, we might expect that all mutations of circadian genes leading to loss of rhythmicity would have a similar phenotype, while in the latter case we can envision how disruption of different circadian genes could have opposite effects.

### Environmental lighting, rhythms and disease

**Figure 2.**
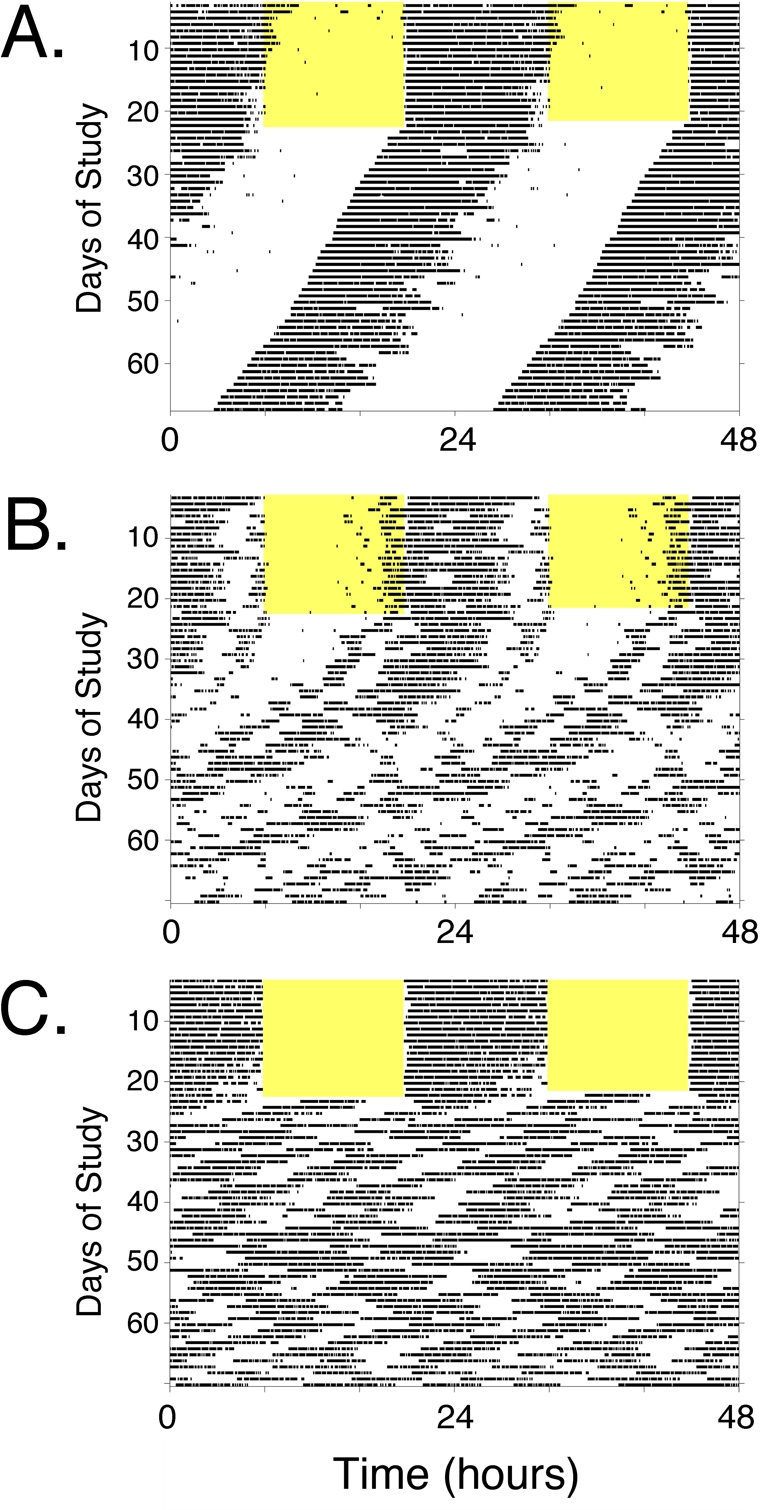
Locomotor activity rhythms of circadian mutant mice. Panels A-C, Double-plotted actograms illustrating types of locomotor activity patterns observed in wild-type and circadian mutant mice (see Tables 1 and 2). Each panel represents the locomotor activity pattern of a single mouse housed in a cage with a running wheel. Periods of voluntary wheel-running appear as dark bars within the grids. Each line of the record shows 48 hours of data across, and successive days are plotted below the first. Each animal was exposed to a standard 12 hour light: 12 hour dark lighting cycle (light period indicated by the yellow shading) for ~ 2 weeks. Then, the light-dark cycle was disabled and the animals were recorded in constant darkness (DD), which allows expression of the animals' endogenous rhythmicity. Visual analysis of the “actogram” allows easy perception of the cycle length (period length, represented by the slope of the line connecting activity onsets) and the robustness of the rhythm when the animal is in DD. (**A**) The actogram in **Panel A** is typical of a wild-type mouse. In the lighting cycle, activity is confined to the dark phase of the cycle. Upon entry into DD, the animal maintains robust rhythmicity with a cycle length of ~ 23.6 hours. This is seen as a leftward shift of activity onset each day by ~ 20 minutes. Lines of mice that would have similar activity records, with or without slight changes in circadian null mutations, CLOCK-deficient mice and NPAS2-deficient mice. Larger alterations in period length but with maintenance of robust rhythmicity is seen in CRY1-deficient mice, CRY2-deficient mice, and *Clock Δ19/+* heterozygotes. (**B**) The actogram in **Panel B** is typical of a mouse with gradual loss of rhythmicity. The light-dark cycle synchronized activity to nighttime, and after transfer to DD, the animal expresses a “free-running” rhythm that gradually decreases in amplitude until arrhythmicity is reached. Mice homozygous for mutation of *Per1* or *Per2* have this phenotype (although in some studies these lines maintain rhythmicity and resemble Panel A). *Clock^Δ19/Δ19^* mutant mice initially have long-period (~ 28 hr) rhythmicity and become arrhythmic within ~ 10 days in constant darkness. (**C**) The actogram in **Panel C** is typical of an animal with complete loss of circadian function. Note that the mouse appears to have rhythmic behavior when exposed to a light-dark cycle, due to suppression of wheel-running activity by ambient light. Following discontinuation of the light-dark cycle, however, the endogenous pattern (or lack there-of) becomes apparent. In this case, the animal immediately loses circa-24-hour rhythmicity. Despite loss of circadian rhythmicity, the animal still has periods of activity and rest, just now these intervals do not have the temporal organization on a 24-hour timescale. Short-period rhythms (4-6 hr cycles) predominate. This pattern is typical of line of mice referred to as arrhythmic, including *Clock/Npas2*-double-knockout (*Clock^−/−^*; *Npas2^m/m^*) mice, *Bmal1^−/−^* knockout, *Per1/Per2* double knockout, and *Cry1/Cry2* double-knockout mice.

It is also worth pointing out that almost all studies of aging-related endpoints in circadian mutant mice have been conducted with animals housed in a light-dark cycle. Rhythmic lighting cycles can drive behavioral and physiological rhythmicity (to varying extents, depending on the genotype and circumstances) even in the absence of a functioning circadian clock (e.g., see Figure 2C). Thus, many studies are assessing the phenotype in the presence of LD- imposed rhythmicity. The phenotype of circadian mutant mice may be considerably more pronounced if they were to be studied after housing in constant dark conditions. (Constant light is rarely used as it can disrupt rhythms if too bright, and controlling light intensity across a group of cages is more difficult than maintaining consistency from cage to cage with darkness.) Two recent studies [48, 83] highlight the exacerbation of that can occur when circadian mutant mice are housed in constant darkness, compared to the same genotype housed in a standard light: dark cycle. Thus, circadian mutant mice are often studied under conditions that may not allow full expression of their genetic deficits. Nevertheless, as both a practical matter related to monitoring of health and with respect to relevance to human health and disease, studying mutant animals in a lighting cycle is appropriate.

Humans, generally, do not reside in constant darkness. Remarkably, however, several studies on cancer incidence among blind individuals reveal a reduced risk [84-90]. Cancer risk is lower when blindness is defined in the strictest sense (those with no capacity for light photoreception), even relative to those that are blind with light perception [80]. Such studies were motivated by the observation that exposure to light at night during shift work leads to circadian disruption and also significantly increases the risk of cancer [86, 87, 89]. Indeed, disrupted circadian rhythmicity caused by light exposure at night has been proposed to underly the increasing prevalence of breast and prostate cancers in industrialized nations. The International Agency for Research on Cancer (IARC) recently concluded that “shift-work that involves circadian disruption is probably carcinogenic to humans” based on “limited evidence in humans” and “sufficient evidence in experimental animals for the carcinogenicity of light during the daily dark period” [91]. Whether these effects are related to nocturnal suppression of melatonin production, as advocated by some, remains to be established; melatonin has anticancer activity in some circumstances [92]. It has been argued that light exposure at night is a predictor of adverse health outcomes, rather than a cause [93].

Several recent studies suggest that the process of repeatedly resetting one's clock is a stressor with adverse effects on health. Repeated exposure to shifts of the light-dark cycle (chronic jet lag) reduces lifespan in aged mice [94], alters the immune response to lipopolysacchiride challenge in young mice [95], accelerates liver carcinogenesis [96-98], and increases susceptibility to gastrointestinal damage by dextran sulfate [99]. Notably, some of these studies were conducted in strains of mice genetically incapable of producing melatonin [100], so shift-induced disruption of the (nonexistent) melatonin rhythm cannot be the mechanism underlying the adverse response. In other studies, lines of hamsters that differ in their free-running cycle length were found to differ in the development of cardiac and renal pathologies; animals housed in a light-dark cycle that did not match their endogenous cycle length had to undergo re-setting of their clock on a daily basis to maintain synchrony to the lighting cycle [101, 102]. Remarkably, in the hamster studies, each line did better when the lighting cycle matched its endogenous cycle length, and poorly (relative to the other line) when there is a mismatch. These animal studies suggest that environmental disruption of circadian rhythms has a significant adverse effect on health, including carcinogenesis and immune function. Circadian misalignment can also affect metabolic function in humans [103] with potential consequences on antioxidant defenses. Thus, there are important consequences of circadian rhythm disruption on aging and cancer.

### Version control

The version of the circadian clock gene hypothesis being tested can vary greatly between studies, and authors are rarely explicit in articulating the version being tested, or its implications. Two extreme cases can make this point. Lee et al., [48] recently proposed that the central circadian clock in the suprachiasmatic nucleus of the hypothalamus coordinates the activity of peripheral clocks via the sympathetic nervous system, and in so doing optimizes anticancer mechanisms within peripheral cells. Their data suggest that any of a number of genetic lesions leading to loss of rhythmicity promote tumorigenesis, e.g., that the circadian clock reduces cancer risk through systemic mechanisms. Furthermore, as noted above, in this study maintaining animals in light-dark cycles masked the cancer phenotype [48]. At the other extreme are data that indicate that altering levels of clock gene expression affects proliferation in vitro; these effects cannot be related to systemic physiological rhythmicity, yet could still involve cellular rhythmicity (which persists in vitro; 104,105]. Data from both types of studies are relevant to the circadian-aging-cancer continuum, but these studies implicate different mechanisms and require different interpretations. Questions relevant to studies of circadian clock genes and cancer include:

- Is behavioral rhythmicity affected by the genetic lesion?
- Is the circadian clock broken in an “on” or “off” state?
- What impact does this gene have on expression of other genes and processes, and are these necessarily related to rhythmicity?

### Circadian clock disruption and aging-related phenotypes

Do the effects of circadian clock gene disruption on aging-related phenotypes make sense with the expectation that all mutations leading to loss of circadian function will have a common phenotype? Or with the competing alternative, that mutations affecting circadian clock function will have effects that make sense based on where the gene falls within the circadian feedback loop? The answer, unfortunately, is no.

#### Disrupting the negative limb of the circadian feedback loop: Per and Cry

Both Per1 and Per2 have been described as tumor suppressor genes [10, 27]. PER2-deficient mice are more susceptible to genetic or radiation-induced cancer [46-48], Per1 or Per2 over-expression reduces tumor growth in vivo and promotes apoptosis in vitro [42-45], while PER down-regulation promotes cancer cell growth [46-48, 52-54 (but see 56, 57)]. Downregulation of Per1 and Per2 gene expression has been reported in human tumors [11, 27, 28, 34-39 (but see 40, 41)]. These studies identify molecular links between PERIOD protein expression and genes known to be involved in apoptosis and cell cycle regulation. These studies might suggest a tumor suppressor role for all proteins that are part of the circadian negative feedback loop. Contrary to this idea, however, is the finding that p53-deficient mice lacking both CRY1 and CRY2 proteins are actually resistant to cancer and have increased lifespan, relative to p53-deficient mice, and CRY1/CRY2-deficiency promoted apoptosis following UV-irradiation [23, 51 (but see 48)]. Thus, paradoxically, CRY deficiency and over-expression of PERs are functionally similar, in suppressing tumor growth. One interpretation for the difference between PERs and CRYs is that the effects of disrupting circadian genes in aging are a complex combination of the contribution of these genes to the circadian clock, along with their role in other cellular processes and pathways [1]. These results, collectively, suggest that there is more going on with circadian mutant lines than “simple” rhythm disruption.

A recent study by Lee et al. [48] examined several mutant mouse models of circadian disruption, and found that virtually all lines of mice examined (including Per2Brdm1 mutants, Cry1-Cry2 double-knockout, *Clock^Δ19/Δ19^*, and *Bmal1*^−/−^, mice) had enhanced tumorigenesis under basal and irradiated conditions. Remarkably, Bmal1 heterozygotes and Cry1-deficient mice, both of which maintain circadian rhythms even in constant darkness, were also more cancer-prone than the controls. The increased cancer incidence in lines that maintain circadian rhythms seems inconsistent with the conclusion that disruption of rhythmic sympathetic outflow underlies the increased tumor development. Furthermore, coat color variation evident in their Figure 1b suggests differences in genetic background between groups.

#### Disrupting the positive limb of the circadian feedback loop: Bmal1 and Clock

The suggestion that there is more going on regarding the clock/aging/cancer interface is reinforced by the results of studies examining genes on the positive side of the transcriptional feedback loop.

Three mouse models have been examined to date (*Bmal1*^−/−^, *Clock*^−/−^, and *Clock*^Δ19/^^Δ19^). The aging and cancer-related phenotypes of these mouse models are not consistent (Table 1), nor are they consistently “opposite” the effects of disrupting the molecular components of the negative feedback loop (Table 2).

The most severe phenotype is observed in BMAL1-deficient mice. BMAL1 is the only gene which, when disrupted, leads to a complete loss of behavioral circadian rhythmicity [106]. Additional phenotypes of BMAL1-deficient mice include greatly reduced lifespan, sarcopenia, age-dependent weight loss, reduced organ weight, cataracts, ectopic calcification of tendons and cartilage, and male and female sterility [50, 107-112]. Restoration of BMAL1 expression in the brain of BMAL1-deficient mice rescued circadian behavioral rhythms but did not prevent ectopic calcification [112]. Rescuing BMAL1 expression in muscle did not restore behavioral rhythmicity or prevent ectopic calcification, but did prevent weight loss [112]. These results clearly reveal that the multiple phenotypes of BMAL-deficient mice cannot all be attributed to the loss of circadian rhythmicity. Instead, BMAL1 expression affects organ function in a site-specific manner, although some systemic influences also are likely [113-115].

While CLOCK-deficient mice have reduced lifespan [1], CLOCK-deficient mice do not have age-dependent reductions in body weight or reduced relative organ weights [1] or ectopic calcification [116], and are fertile (although female reproductive performance is reduced; our unpublished results). In these animals, behavioral rhythms are preserved, while peripheral tissues have only systemically-driven rhythms, indicating that peripheral circadian oscillators require CLOCK [117, 118].

CLOCK-deficient mice also appear to differ, phenotypically, from *Clock^Δ^*^19/^*^Δ^*^19^ mutant mice, which have an intronic splice site point mutation in the Clock gene that leads to exclusion of exon 19 from the transcript. This mutant CLOCK protein (CLOCKΔ19) is devoid of transcriptional activity but can bind to BMAL1; from biochemical and genetic evidence, the mutant protein appears to function as a dominant negative [4, 5, 119, 120]. *Clock^Δ^*^19/^*^Δ^*^19^ mutant mice have a modest aging/cancer phenotype [49; Table 1]. Lifespan under routine conditions is unaffected [Antoch, unpublished data, cited in [49]]. Radiation-induced body weight loss, organ weight loss, and lifespan shortening are more pronounced in female *Clock^Δ^*^19/^*^Δ^*^19^ mutant animals, relative to wild-type controls. In contrast, male *Clock^Δ^*^19/^*^Δ^*^19^ mutant mice do not differ from controls in lifespan after irradiation (although they did have greater body weight loss than controls after irradiation) [49]. Female *Clock^Δ^*^19/^*^Δ^*^19^ mutant mice have reduced fecundity and are prone to dystocia, but remain fertile [121-122], in contrast to the sterility of both sexes observed in BMAL1-defient mice [106, 109, 110] and in *Clock*^−/−^;*Npas^2m/m^* double-mutant [unpublished data].

With respect to aging phenotypes in the absence of irradiation, the mutant lines can be ordered in decreasing severity as follows: *Bmal1*^−/−^ >> *Clock*^−/−^ > and *Clock^Δ^*^19/^*^Δ^*^19^. It will be of considerable interest to see if the radiation response of *Clock^−/−^* mice is similarly intermediate between these other two mouse lines with disruption of the circadian transcriptional activation complex.

It is somewhat remarkable that *Clock^Δ^*^19/^*^Δ^*^19^ mutant mice do not appear to be as severely affected as the CLOCK-deficient mice with respect to lifespan. *Clock^Δ^*^19/^*^Δ^*^19^ mutant mice on the C57BL/6J background lose locomotor activity rhythmicity when housed in constant darkness [120, 123], while *Clock*^−/−^ mice maintain behavioral rhythmicity through the redundant action of NPAS2 in the SCN [4, 5, 117]. Tissues explants and fibroblasts from all three mutant lines (*Clock*^−/−^,*Clock^Δ^*^19/^*^Δ^*^19^, and *Bmal1*^−/−^) lose rhythmicity, while tissues from wild-type mice maintain rhythms in vitro [118, 124, 125]. If disruption of circadian oscillations leads to acceleration of aging through disruption of the synchronization of metabolic processes [126], one might expect that all three lines of mice, with equally “incompetent” peripheral oscillators, would have equal phenotypes. They do not. Of these lines, the *Clock*^−/−^ mice appear to have the best circadian clock function: they can maintain behavioral rhythmicity in constant darkness, while the other lines cannot (Figure 2). While many holes remain to be filled in Tables 1 and 2, from these studies it appears there is not a good correlation between the extent of circadian rhythm disruption and the aging/cancer phenotype.

### Prospectus

What does the future hold for studies of the role of circadian clock genes in aging, response to genotoxic stress, and cancer? Most obviously, filling in the gaps in Tables 1 and 2 is needed. How are the acute response to radiation and radiation-induced morbidity, cancer incidence, and mortality influenced by the absence of CLOCK? What are the causes of premature death in CLOCK-deficient mice? A striking preliminary conclusion from the studies discussed here is that lifespan and cancer incidence of circadian mutant lines under baseline conditions appear to be poorly predictive of each other or of responses to genotoxic stress and irradiation [22, 33, 49, 50].

Another line of investigation is to understand the molecular mechanisms underlying the age-related pathologies in CLOCK-deficient mice identified by Dubrovsky et al [1]. What does a higher incidence and earlier onset of dermatitis in CLOCK-deficient mice mean? Differences in aggression and interactions between cage-mates or in grooming could contribute [1], but in addition, the accelerated, age-dependent incidence of dermatitis in CLOCK-deficient mice could be due to loss of an important, local action of the CLOCK:BMAL1 dimer and/or circadian rhythms of gene expression. Fibroblasts, including human dermal fibroblasts, have tissue-autonomous molecular circadian rhythms [124, 125, 128-132]. Rhythmic clock gene expression in skin is disrupted in CRY-deficient or SCN-lesioned mice [133]. Hair follicle synchrony (on a ~ 3-week cycle length) is altered in CLOCKΔ19 and *Bmal1^−/−^* mice [134, 135], further indicating a role for circadian clock function in skin. Thus, disruption of circadian clock genes likely disrupts skin physiology by local actions. Alternatively, systemic hormonal, metabolic or nutritional factors may influence dermal integrity. Histological examination and assessment of constituent cell populations is necessary to relate the observation of increased dermatitis incidence in CLOCK-deficient mice to epithelial, mast, and dendritic cells, sebaceous glands and hair follicles.

Similarly, what does the higher incidence of cataracts in CLOCK-deficient mice mean? Cataracts are often taken as an indication of excessive reactive oxygen species (ROS) and oxidative stress, and indeed, in BMAL1-deficient mice, cataracts are delayed by treatment with an antioxidant [107]. Does the increased age-dependent incidence of cataracts in CLOCK-deficient mice reflect whole-body ROS status, or local events such as oxygen tension and ion transport in the lens epithelia? In future studies, it may be possible to distinguish systemic, metabolic influences from local effects by local manipulation of these genes, using conditional alleles of these genes (in which critical exons are flanked by loxP sites) and tissue-specific drivers for Cre recombinase. Tissue-specific gene disruption is beginning to be employed in studies of circadian rhythms [78, 82, 114, 115, 117].

When discussing circadian rhythms and oxidative stress, melatonin may come to mind. As noted above, melatonin is produced in a circadian rhythm, with levels elevated at night, and the hormone is widely investigated with respect to its antioxidant properties following administration at pharmacological levels [136, 137]. The role of endogenous melatonin in antioxidant defense is unclear. In the present context, however, it is clear that melatonin can play no role: C57Bl/6J mice do not produce pineal melatonin [100].

Another interesting area for study will to be to determine the extent to which NPAS2 substitutes for CLOCK, mitigating the impact of CLOCK deficiency (relative to the more profound effects of BMAL1-deficiency). In the SCN, NPAS2 maintains circadian rhythmicity in the absence of CLOCK [4]. In contrast, peripheral tissues are unable to maintain rhythmicity in the absence of CLOCK [111, 138, but see 139]. Mice lacking both CLOCK and NPAS2 have a profound phenotype, appearing very similar to mice lacking BMAL1 with respect to age-dependent weight loss, arthropathy, male and female sterility, and premature death [Table 1; our laboratory's unpublished data]. The very profound accelerated aging phenotype of these double-knockout mice, especially when compared to mice lacking CLOCK alone, suggests an important, redundant contribution of NPAS2—but where? In CLOCK-deficient mice, pathologies may be restricted to those tissues that have naturally low levels of NPAS2 expression, so in these tissues there is a breakdown of redundancy. Alternatively, the loss of systemic rhythmicity may interact in important ways. An exciting prospect is that the conditional (floxed) alleles of Clock (in an NPAS2-deficient background) or conditional alleles of Bmal1 will allow identification of sites and mechanisms of action of premature spontaneous aging and of accelerated aging induced by genotoxic stress. Furthermore, the availability of mouse lines in which expression of CLOCK or BMAL1 can be induced with anatomical and temporal control [112, 140] allow complementary genetic manipulations to gene disruption strategies.

## CONCLUSIONS

While the studies conducted to date do not, in our opinion, generate a clear conclusion about the contribution of circadian rhythmicity to normal aging, several circadian clock genes are clearly important in regulating lifespan, cancer susceptibility and the cell cycle. Further studies involving manipulation of circadian clock genes, and manipulating the environmental housing conditions in which animals are studied, have much to offer in the study of aging.
